# Factors affecting expert performance in bid evaluation: An integrated approach

**DOI:** 10.3389/fpsyg.2022.819692

**Published:** 2022-08-04

**Authors:** Li Wang, Kunhui Ye, Yu Liu, Wenjing Wang

**Affiliations:** ^1^School of Management Science and Real Estate, Chongqing University, Chongqing, China; ^2^School of Civil Engineering, Architecture and Environment, Xihua University, Chengdu, China; ^3^International Research Center for Sustainable Built Environment, Chongqing University, Chongqing, China

**Keywords:** expert performance, cognitive psychology, situation perception, supervision, MICMAC-ISM approach

## Abstract

Experts play a crucial role in underpinning decision-making in most management situations. While recent studies have disclosed the impacts of individuals’ inherent cognition and the external environment on expert performance, these two-dimensional mechanisms remain poorly understood. In this study, we identified 14 factors that influence expert performance in a bid evaluation and applied cross-impact matrix multiplication to examine the interdependence of the factors. The results indicate that the two dimension-related factors affect each other within a person–environment system, and a poor situation perception gives rise to the deviation of expert performance. Expert performance can be improved if external supervision and expertise are strengthened through deliberate practices. The study proposes a new expert performance research tool, elucidates its mechanism in bid evaluation from a cognitive psychology perspective, and provides guidelines for its improvement in workplace contexts.

## Introduction

One of the most heated research issues in management science is decision-making, which is strongly associated with experts’ scientific and accurate judgment (Bolger, 1996). Prior research has affirmed that the advice and judgment of experts are prominent in situations with limited data, enormous uncertainty, timely solutions, and unpredictable trends (Burgman et al., 2011). For example, there is a need for expert-aided decision-making in natural resource management to assess hazards (Victoria et al., 2018). In this sense, expert performance is highlighted to describe the process of providing quality services to meet societal demands. An expert’s satisfactory performance depends on whether the expert has sufficient expertise, qualifications, professional morality, and experience. For simplicity, Mcpherson and Kernodle (2007) called these factors “inherent cognition.”

However, given inherent cognition, experts may not provide quality judgments. Havers et al. (2019) suggested that the reason could be the conflicts of stakeholders’ interests and relationships. As a result, the expert is intended to make a biased decision and causes poor performance. Recent studies have further argued that personal reputation and fatigue deserve closer attention as they misguide experts to make incomplete decisions in the peer review process (Rodriguez et al., 2007; Burgman et al., 2011). Experts advocate balancing motivational and external constraints (e.g., external pressure, scenario perception, institutional systems; Ericsson et al., 1993). Thereby, expert performance can be improved. According to Araújo et al. (2006), such factors are external and related to material and social environments.

The synthesis of inherent cognition and external environmental factors suggests that expert performance is dynamic, systematic, interactive, and characterized by multiple feedbacks. As ecological cognition theory elaborates, experts prefer to build their decisions on the interaction between inherent cognition and external environmental factors (Vanda et al., 2013). Victoria et al. (2018) argued the protocol for structured expert elicitation is more conducive to coping with external impacts on inherent cognition than randomly capturing changes in dynamic environments. Therefore, attempts to promote expert performance should consider the interaction between inherent cognition and the external environment.

The enhancement of expert performance is fueled by an increase in inherent cognition and the ability to adapt to the external environment. However, there is scant research on the interaction between inherent cognition and the external environment (Ericsson, 2007; Baker et al., 2010; Li et al., 2014; Helfrich et al., 2018). It is also unsure about how inherent cognition and external environmental factors advance expert performance and how these factors drive one another in an industrial setting. This study aims to bridge this knowledge gap by examining expert performance-related factors. The research objectives are to identify the key factors of expert performance in bid evaluation and evaluate the interrelationships between the identified factors using a driving/dependence power graph. Our findings shed some light on a shift of expert performance concepts from cognitive to management science. Furthermore, we laid a foundation for future studies to generalize expert performance determinants, considering the uncertainty of environmental factors rather than merely psychological representations and cognitive scientific calculations.

## Literature review

### Rethinking expert performance definitions

Expert performance was coined as a key construct for theorizing expert performance (Ericsson, 2007; Debarnot et al., 2014; Hashimoto et al., 2015). Experts are engaged in providing knowledge-intensive professional services for complex questions. Traditionally, a closed system perspective is adopted to interpret experts’ long-time superior performance (Ericsson, 2008; Ericsson and Harwell, 2019). The long-time superior performance is tied to the accumulation of deliberate practices and feedback that they encode and the refinery of inherent representations memorized for effective use in a determinate world (Ericsson and Kintsch, 1995). Similarly, novices may be likely to produce opinions and judgments as experts do for the same matters if they have enough knowledge and experience (Spence, 1993). As revealed in scientific judgment and the improvement of learning patterns (Krueger et al., 2012; Campitelli et al., 2015; Friedman and Korman, 2019), inherent cognition, including knowledge and experience, pertains to the generation of expert performance. In a closed system, experts’ performances are evaluated without considering the fluctuations in situations they are working with. Unexpected emergencies are precluded in the determination of expert performance.

However, the “closed system” perspective is subject to considerable flaws, as indicated by quantum physics and social and psychological science (Glimcher, 2005). Researchers have thus claimed to advance the closed system to embrace indeterminacy in the research area of brain and behavior (Gigerenzer et al., 2000; Hastie, 2001; Schall, 2004). An open system is consequently framed. Based on the tenet of the open-system philosophy, the determinants of expert performance are beyond the boundary of inherent cognition. For example, an expert’s performance does not necessarily exceed that of a novice, regardless of whether the problem is relatively simple or complicated. Chi (2006) and Spence (1993) indicated that inherent cognition might not realize better performance in judgment and decision making as experts must adapt to a new environment. Over the years, with the development of ecological cognitive science, Araújo et al. (2006) proposed an effective way to obtain superior expert performance by capturing specific environmental and perceptual information. Therefore, improving perceptions about environmental factors and owning privileged access to refined inherent representations are crucial to expert performance (Ericsson and Kintsch, 1995).

### Expert performance in bid evaluation

When decision-making time and support resources are stretched, the external environment becomes a predominant factor in the formation of expert performance. This is the case in competitive bidding in the construction sector, where experts are highly involved in evaluating submitted bids. As a result, experts must produce professional services to underpin the determination of winners in competitive bid evaluations. To address the issue, previous studies have proposed approaches and models to aid experts in evaluating bids (Liu et al., 2017; Semaan and Salem, 2017). However, they often face inadequate information and uncertain circumstances (Zhen-Song et al., 2021), suggesting that the interaction between inherent cognition and the external environment is not monotonic.

Previous studies have offered a few approaches to examining the links between inherent cognition and the external environment, such as measuring eye movements and verbal reports (Macmahon and McPherson, 2009; Afonso et al., 2012). The approaches address bid evaluation task constraints and behavior settings in experimental. However, laboratory-based simulations represent a real-life situation where experts are vulnerable to biases in decision-making (Araújo et al., 2006). Notwithstanding the complex factors of bid evaluation, expert performance has been paid extensive attention, Kardes (2006), Burgman et al. (2011), and Connor et al. (2020) proposed that it was better to redefine expert performance to account for those contextual and relational factors of expert performance.

## A conceptual framework

### Person–environment system

**Figure 1:**
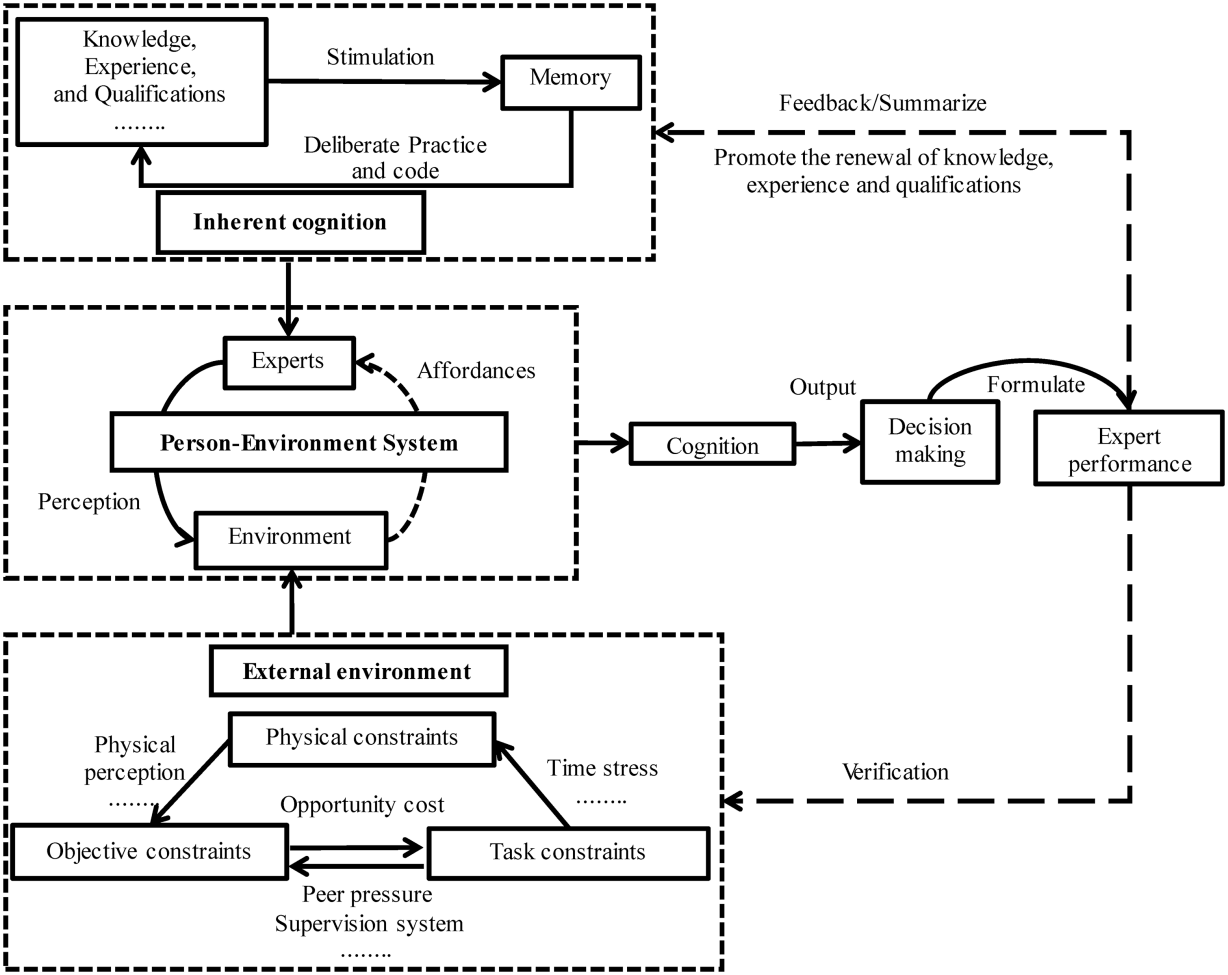
The formation mechanism model of expert performance in bid evaluation.

According to the open-system concept, we outlined two dimensions of expert performance: inherent cognition and the external environment. A conceptual framework is proposed below to describe the mechanism of expert performance (Figure 1).

As Figure 1 tells, the formation mechanism of expert performance contains two parts: memory and ecological psychology. Inherent cognition is grounded on theories of memory, of which the main components are knowledge, experience, and qualifications (Ericsson, 2003). The formation of inherent cognition contains a loop in which knowledge, experience, and qualifications give a stimulus to memory. A changed memory enlarges the packet of knowledge, experience, and qualifications. This loop mirrors the formation of inherent cognition (Ericsson, 2007). To align with the enlargement, deliberate practice, encoding, and transformation transform external stimuli into meaningful representations (Kording and Wolpert, 2006). Meanwhile, ecological psychology is referred to explain the impacts of the external environment on expert performance, assuming a person–environment system (PES; Araújo et al., 2006). As Turvey et al. (1981) pointed out, actions, judgment, and performance are based on the lawful connections between individuals and the environment in which they act. Therefore, experts are engaged in social culture, rules, and regulations belonging to the external environment.

Experts utilize external environmental factors to avoid biased decisions (Fischhoff et al., 1982; Ludger and Erik, 2016; Contreras-Pacheco et al., 2020). However, the factors also pose physical, goal-related, and task-related constraints to expert performance (Araújo et al., 2006). These constraints cause experts to feel physical discomfort (Liu et al., 2015), peer pressure (Bohannon, 2013), motivation deviation (Ericsson et al., 2009), loose supervision (Gonsalvez et al., 2017), and weak feedback (Kardes, 2006). Furthermore, given a specific external environment, expert performance may combine inherent cognition with the external environment to present a holistic “person–environment system.” Expert performance results from dynamic, continuous interaction within the PES, which is influenced by inherent cognition and external environmental perceptions.

### Interaction between PES and expert performance in bid evaluation

According to ecological psychology theory, bid-evaluation experts’ inherent cognition and perception of the external environment are embedded in each other, emphasizing that the interaction between experts and the environment is an important motivation for expert performance. In the PES, bid-evaluation experts have many opportunities to address the opportunities or possibilities for actions, known as *affordances,* and seize a set of objective and physical external environmental factors to improve their performance (Costall, 1984). Regarding bid-evaluation experts, perceiving an “*affordance*” means perceiving how one can make decisions under specific bid evaluation conditions (Araújo et al., 2019).

In the bid evaluation process, experts are supposed to integrate multiple attributes into the overall description of bidders. This is a perceptual process in which bidders’ redundant and irrelevant attributes are removed, and their core attributes are framed. Finally, the experts form inherent cognitive “representations” of the bidders. Interweaving in the environment and perceptual processes (from reactions to relevant external objects and events), this cognitive process is dynamic. Therefore, a bid evaluation task is one in which “*affordance*” captures the interaction between experts and the environment (e.g., peer pressure, time pressure, supervision system, and opportunity cost) with a concatenation of interdependent decisions over time. At the end of bid evaluations, experts provide feedback by reflecting on or summarizing the results from the external environment to change inherent cognition. For example, whether a bid award decision is passable is determined not by its absolute attribution representation (whether measured in performance, qualification, or scale) but rather by how it relates to the substantive responses of an individual bidder to the rules, including technical requirements, economic indicators, and commitments.

## Research methods

Expert performance impacts have been examined by exploratory research, including conceptual research (Ericsson and Ward, 2007) and case analysis (Araújo et al., 2006), with a focus on the influence of inherent cognitive or external environmental factors. However, based on experimental research methods, most quantitative research designs lead to the emergence of artificial decisions and behaviors (Sozzo, 2020). Therefore, we adopted a new perspective by investigating the determinants of on-the-job performance of experts within specialist domains in the workplace. First, two-dimensional factors: inherent cognition and the external environment, were identified. Second, we detected expert performance factors through a literature review. Third, in-depth interviews with experts were implemented to confirm the reliability of the factors. Fourth, factors were refined based on the experts’ evaluations of the similarity and necessity of the factors. Finally, we compiled a list of bid-evaluation expert performance factors. Consequently, the applicability and usefulness of the factors to attain a hierarchical structural framework were confirmed.

### Interpretative structural modeling

**Figure 2:**
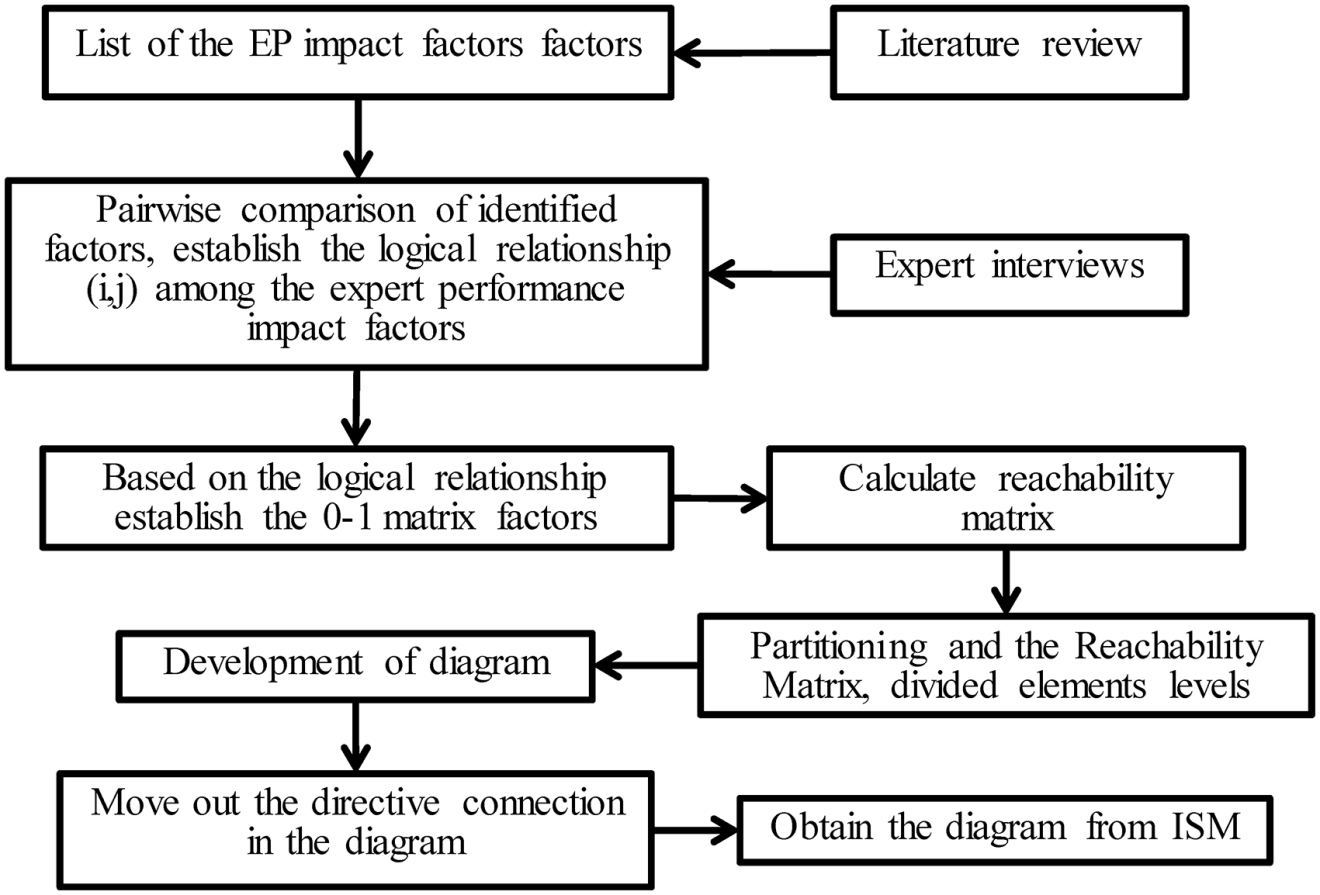
The technical route of interpretive structural modeling.

We detected these factors’ dependency/driving power using cross-impact matrix multiplication, which is often applied to classification and interpretative structural modeling (MICMAC-ISM). Warfield (1974) established a computer-assisted learning process called interpretive structural modeling (ISM) to transform unclear and poorly articulated mental models into well-defined multi-level structural models through experts’ practical experience and knowledge. As many interrelated factors affect expert performance in bid evaluation, we selected ISM for our research methodology. The reason not only goes to a well-established methodology for identifying relationships among specific items but also to providing a fundamental understanding of complex situations. Therefore, this study established an ISM-based hierarchical structure model to clarify the dynamics of relationships among expert performance factors encountered in the bid evaluation practices. The technical route of the ISM is shown in Figure 2.

### Expert performance factors in bid evaluation

Following the literature review, we categorized expert performance dimensions into 16 factors. A questionnaire was designed, and 34 experts (from construction, design, cost consulting enterprises, research institutions, and government departments) were interviewed to test the reliability of these dimensions and factors. The interviewees were selected based on their bid-evaluation qualifications and experience, and a deep understanding of construction contracts. The profiles of the interviewees are given in Table 1. To allow them to understand the relationships among the factors, we conducted face-to-face interviews with all the experts. As a result, three factors were removed, and a new one was added. Finally, the remaining 14 bid-evaluation expert performance factors were compiled. A summary of these factors is presented in Table 2.

**Table 1:** Identification of expert performance factors per dimension.

	Code	Factors	Definitions	References
Inherent cognition	*S_1_*	Expertise of bid evaluation	The expertise is the education background and major of bid evaluation experts	Thompson et al., 2013; Hambrick et al., 2014
	*S_2_*	Academic ability of the bid evaluation experts	The ability to solve practical bid evaluation problems with the knowledge learned, the reaction speed, learning ability and observation ability when encountering the related cases, etc.	Ericsson, 2014; Hamdi et al., 2017
	*S_3_*	Motivation preference	Theoretical research or social practice	Ericsson et al., 2009; Hannele et al., 2016
	*S_4_*	Years qualified as a bid evaluation expert	Proficiency of practice and experience of bid evaluation experts’	Lund et al., 2017; Erturan et al., 2018
	*S_5_*	The number of bid evaluations	Practical experience	Fabian et al., 2016
	*S_6_*	Morality of bid evaluation experts	Is there any record of bid evaluation violation due to behavior bias	Lee and Ryzin, 2019
	*S_7_*	Objectivity of bid evaluation experts	Bidding documents and understanding thereof	Wang et al., 2013; Wei et al., 2019
External environment	*S_8_*	Situation perception of bid evaluation expert in the workplace	Physical environment in the bid evaluation workplace (sitting comfort, air humidity, seat position, physical comfort)	Foglia and Wilson, 2013; Liu et al., 2015
	*S_9_*	Supervision system of bid evaluation	Politics, Laws, Regulations of bid evaluation	Gonsalvez et al., 2017
	*S_10_*	Stress situation of bid evaluation expert	Responsibilities and Task-related constraints of bid evaluation experts From peer pressure or Coercion	Michaels, 2000; Whitehead et al., 2016
	*S_11_*	Natural environment of bid evaluation	Season (seasonal characteristics can cause mood changes among bid evaluation experts), Climate (storms, snow, heat, typhoons, and other extreme weather), etc.	Heyden, 2013; Hambrick and Tucker-Drob, 2015
	*S_12_*	Distance of bid evaluation	Distance from bid evaluation site	Caset et al., 2019; Jo et al., 2019
	*S_13_*	Strength of bid evaluation	Bid evaluation rounds Responsibility Target Time constrained	Michaels, 2000; Lindfield and Penny, 2012
	*S_14_*	Rewards of bid evaluation	Goal-related constrains (charge, honors for bid evaluation experts, etc.)	Fuller et al., 2013; Korhonen-Sande and Sande, 2016

**Table 2:** Background profiles of interviewed experts.

Category	Classification	Number of experts	%
Education background	Bachelor’s Degree	19	56%
	Master’s Degree	9	26%
	PhD	4	12%
	Others	2	6%
Job profile/department	Construction enterprise	12	35%
	Design enterprise	3	9%
	Cost consulting enterprise	4	12%
	Research institution	9	26%
	Government departments	3	9%
	Others	3	9%
Years of expert qualification(experience)	1 ~ 5 years	10	29%
	6 ~ 10 years	6	18%
	Over 10 years	18	53%

### Generation of the adjacency matrix A

The relationships among the 14 factors were confirmed *via* interviews with 34 experts. Using ISM, a “leads to” (one factor leading to another) contextual-type relationship was chosen to identify the interacting position of each factor for analysis. An adjacency 14*14 matrices of the identified impact factor elements (1 = Yes; 0 = No) was developed as an option for paired comparison between elements. The presence or absence of a relationship directed from element *i* to *j* was indicated by placing “1” or “0,” respectively, in the corresponding intersection of row *i* and column *j*. After that, information was sent to experts for comments. Based on their responses, we used the Delphi technique to obtain common views from the participants. When 80% of the interviewees agreed upon the relation, it was thought to have a consensus. The threshold value for the reliability of factors was 0.80. Adjacency reachability matrix A, indicating the relationship between elements, is presented in Table 3.

**Table 3:** Adjacency reachability matrix A of expert performance factors.

Code	*S_1_*	*S_2_*	*S_3_*	*S_4_*	*S_5_*	*S_6_*	*S_7_*	*S_8_*	*S_9_*	*S_10_*	*S_11_*	*S_12_*	*S_13_*	*S_14_*
*S_1_*	0	0	0	0	1	0	0	0	0	0	0	0	0	0
*S_2_*	1	0	0	1	0	0	0	0	0	0	0	0	0	0
*S_3_*	1	1	0	0	0	0	0	0	0	0	0	0	0	0
*S_4_*	1	0	1	0	1	0	0	0	0	0	0	0	0	0
*S_5_*	1	0	0	0	0	0	0	0	0	0	0	0	0	0
*S_6_*	0	0	1	0	0	0	0	0	0	0	0	0	0	0
*S_7_*	1	0	1	0	0	0	0	0	0	0	0	0	0	0
*S_8_*	0	0	1	0	1	0	0	0	0	0	0	0	1	0
*S_9_*	0	0	0	0	0	1	0	0	0	0	0	0	0	0
*S_10_*	0	0	0	0	0	0	0	0	0	0	0	0	0	0
*S_11_*	0	0	0	0	0	0	0	0	0	0	0	0	0	0
*S_12_*	0	0	0	0	0	0	0	0	0	0	0	0	0	0
*S_13_*	0	0	0	0	0	0	0	0	0	0	0	0	0	1
*S_14_*	0	0	0	0	1	0	0	0	0	0	0	0	0	0

### Reachability matrix calculation

From the adjacency reachability matrix A, redundant relationships between elements were eliminated through an iterative process. A diagram established the final relationship level between elements in a hierarchical form. As the adjacency reachability matrix A is a Boolean matrix, we calculated it by applying the following Boolean algorithm:

$$\mathrm{let}\;I\left(n\times n\right)=\left[\begin{array}{cccc} 1 & 0 & \cdots & 0\\ 0 & 1 & \cdots & 0\\ \vdots & \vdots & \ddots & \vdots \\ 0 & 0 & \cdots & 1. \end{array}\right]$$

(1) $$R={\left(A+I\right)}^{\left(n+1\right)}={\left(A+I\right)}^{n}\neq \cdots \neq {\left(A+I\right)}^{2}\neq \left(A+I\right)$$

where $R={\left(A\;\;+\;\;I\right)}^{n}$ is the reachability matrix R of matrix A.

According to adjacency reachability matrix I of the bid-evaluation expert performance, the calculation process was implemented in MATLAB (Lindfield and Penny, 2012). When n=5 , the equation $\;M={\left(A+I\right)}^{\left(n+1\right)}={\left(A+I\right)}^{n}\neq \cdots \neq {\left(A+I\right)}^{2}\neq \left(A+I\right)$ held. The derived result and the reachability matrix are shown in Table 4.

**Table 4:** Reachability matrix of expert performance factors.

Elements *S*(*i/j*)	*S*_1_	*S_2_*	*S_3_*	*S_4_*	*S_5_*	*S_6_*	*S_7_*	*S_8_*	*S_9_*	*S_10_*	*S_11_*	*S_12_*	*S_13_*	*S_14_*	Driving power
*S_1_*	1	0	0	0	1	0	0	0	0	0	0	0	0	0	2
*S_2_*	1	1	1	1	1	0	0	0	0	0	0	0	0	0	5
*S_3_*	1	1	1	1	1	0	0	0	0	0	0	0	0	0	5
*S_4_*	1	1	1	1	1	0	0	0	0	0	0	0	0	0	5
*S_5_*	1	0	0	0	1	0	0	0	0	0	0	0	0	0	2
*S_6_*	1	1	1	1	1	1	0	0	0	0	0	0	0	0	6
*S_7_*	1	1	1	1	1	0	1	0	0	0	0	0	0	0	6
*S_8_*	1	1	1	1	1	0	0	1	0	0	0	0	1	1	8
*S_9_*	1	1	1	1	1	1	0	0	1	0	0	0	0	0	7
*S_10_*	0	0	0	0	0	0	0	0	0	1	0	0	0	0	1
*S_11_*	0	0	0	0	0	0	0	0	0	0	1	0	0	0	1
*S_12_*	0	0	0	0	0	0	0	0	0	0	0	1	0	0	1
*S_13_*	1	0	0	0	1	0	0	0	0	0	0	0	1	1	4
*S_14_*	1	0	0	0	1	0	0	0	0	0	0	0	0	1	3
Dependence power	11	7	7	7	11	2	1	1	1	1	1	1	2	3	56

### Reachability matrix R analysis

The reachability matrix obtained above was partitioned by deriving the reachability set and the antecedent set to establish the hierarchy model of the ISM. The reachability set for each element represented a set of elements (i.e., several risk elements, including itself) upon which the current element had an impact. For example, in the ith horizontal row *Si* of reachability matrix R, if $S_{ij}=1\left(j=1,2,\cdots ,n\right)$ , the element S*ij* is placed in the reachability set, expressed as S*i*. The antecedent set reflects a set of elements that affected the current element. Along the same lines, in the jth column S*j* of reachability matrix R, if $R_{ij}=1\left(i=1,2,⥂\cdots ,n\right)$ , the element R*ij* is placed in the antecedent set, expressed as A*j*. The intersection of these sets $S_{i}\cap A_{j}$ was derived for all elements. If S*i* is a complete subset of A*j*, the element or elements were moved from the reachability matrix and assigned a specific level.

After the iteration, the reachability set for the elements Expertise (S1) and Number of bid evaluations (S5) was a complete subset of the antecedent set; therefore, it was considered the highest level of the elements removed from the reachability matrix. On the other hand, the elements Stress situations (S10), Natural Environment (S11), and Distance (S12) occupied the reachability sets and the antecedent sets on their own, which implies that these factors were isolated from other elements. They were, therefore, removed from the reachability matrix and placed on the first level. The iterative processes were continued in this manner to identify the different levels. Consequently, the hierarchy model of the ISM consisted of all these levels. The results of the final iteration are given in Table 5.

**Table 5:** Level partition of reachability matrix.

Elements *S*_(*i/j*)_	Reachability set:R(Si)	Antecedent set:A(Si)	Intersection R(Si)∩A(Si)
Level I = {S_1_, S_5_, S_10_, S_11_, S_12_}			
*S_1_*	1, 5	1, 2, 3, 4, 5, 6, 7, 8, 9, 13, 14	1, 5
*S_2_*	1, 2, 3, 4,5	2, 3, 4, 6, 7, 8, 9	2, 3, 4
*S_3_*	1, 2, 3, 4, 5	2, 3, 4, 6, 7, 8, 9	2, 3, 4
*S_4_*	1, 2, 3, 4,5	2, 3, 4, 6, 7, 8, 9	2, 3, 4
*S_5_*	1, 5	1, 2, 3, 4, 5, 6, 7, 8, 9, 13, 14	1, 5
*S_6_*	1, 2, 3, 4, 5, 6	6, 9	6
*S_7_*	1, 2, 3, 4, 5, 7	7	7
*S_8_*	1, 2, 3, 4, 5, 8, 13, 14	8	8
*S_9_*	1, 2, 3, 4, 5, 6, 9	9	9
*S_10_*	10	10	10
*S_11_*	11	11	11
*S_12_*	12	12	12
*S_13_*	1, 5, 13, 14	8, 13	13
*S_14_*	1, 5, 14	8, 13, 14	14
Level II = {S_2_, S_3_, S_4_, S_14_}			
*S_2_*	2, 3, 4	2, 3, 4, 6, 7, 8, 9	2, 3, 4
*S_3_*	2, 3, 4	2, 3, 4, 6, 7, 8, 9	2, 3, 4
*S_4_*	2, 3, 4	2, 3, 4, 6, 7, 8, 9	2, 3, 4
*S_6_*	2, 3, 4, 6	6, 9	6
*S_7_*	2, 3, 4, 7	7	7
*S_8_*	2, 3, 4, 8, 13	8	8
*S_9_*	2, 3, 4, 6, 9	9	9
*S_13_*	13, 14	8, 13	13
*S_14_*	14	8, 13, 14	14
Level III = {S_6_, S_7_, S_13_}			
*S_6_*	6	6, 9	6
*S_7_*	7	7	7
*S_8_*	8, 13	8	8
*S_9_*	6, 9	9	9
*S_13_*	13	8, 13	13
Level IV = {S_8_, S_9_}			
*S_8_*	8	8	8
*S_9_*	9	9	9

### Development of a diagram

**Figure 3:**
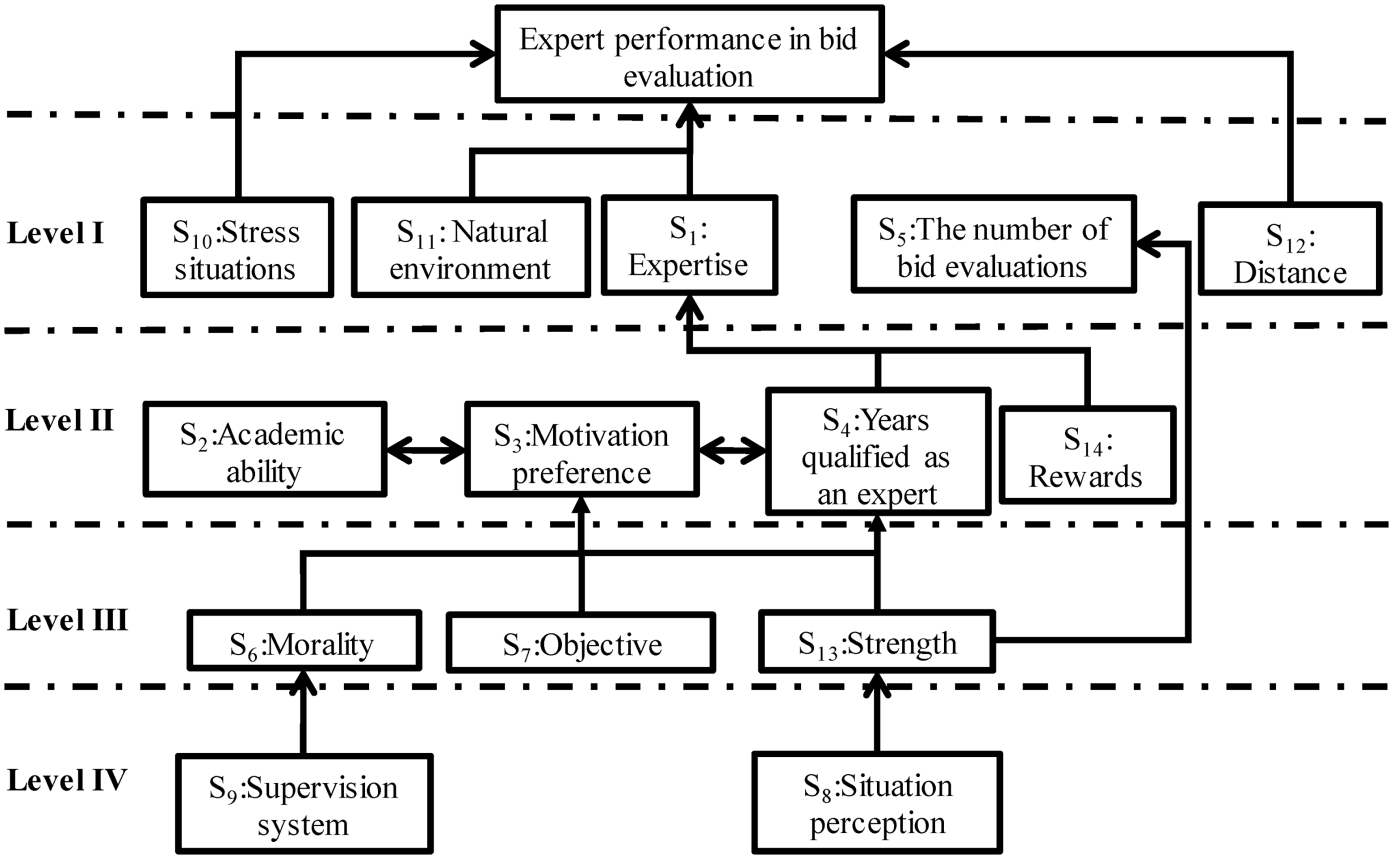
Diagram of expert performance factors in bid evaluation.

A diagram explains the contextual relationship between an impact factor element and its hierarchy. First, as shown in Table 5, the elements taken from the reachability matrix in the previous step were placed at the highest level of the hierarchy. Thus, Expertise (S1), Number of bid evaluations (S5), Stress situations (S10), Natural environment (S11), and Distance (S12) appeared at the top. Next, the elements of Academic ability (S2), Motivation preference (S3), Years qualified as an expert (S4), and Rewards (S14) were removed before the next partition at the second level and placed below the top level. This process was repeated until all the elements were rearranged, creating a four-layer hierarchical structural diagram of the expert performance factors in bid evaluation (Figure 3).

**Figure 4:**
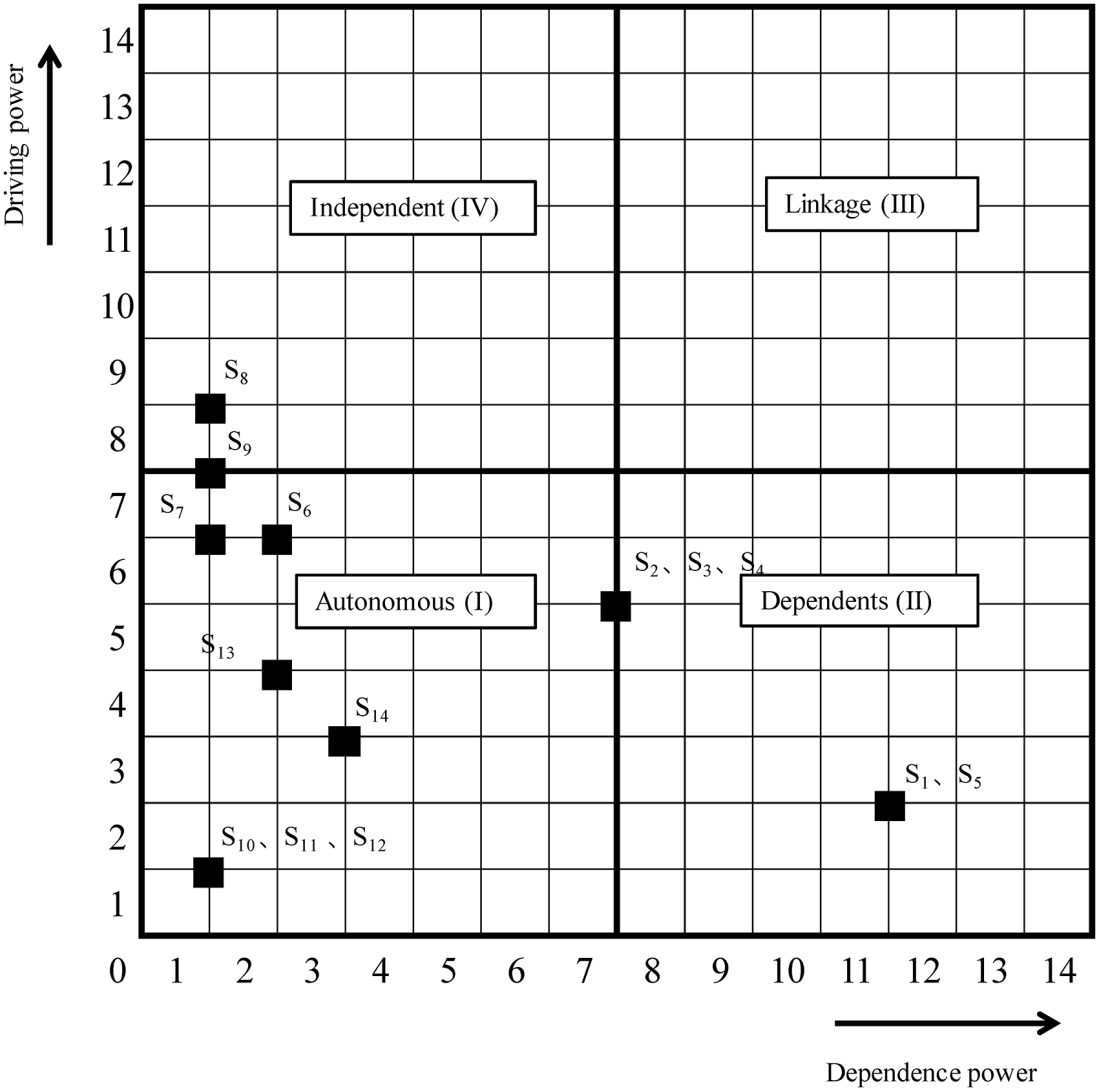
Classification of factors.

Using MICMAC analysis, it is necessary to prove the relationships among impact factor elements based on the attained diagram. We used this approach to analyze a factor’s dependency and driving power. The analysis complements experienced users’ impressions drawn from the visual analysis of influence structures. To better understand the significance of certain elements and their influence on others, we used the MICMAC to classify the factors into four clusters (autonomous, dependent, linkage, and independent) according to their driving and dependence power. The driving-and-dependence power of an element was computed *via* the summation of the corresponding rows. Similarly, the dependence power was computed *via* the summation of the corresponding rows and columns, respectively. Finally, each element was plotted on the driving-dependence power matrix (Figure 4).

The first cluster represents “autonomous factors” (weak driving and dependence power). It includes Stress situations (S10), Natural environment (S11), Distance (S12), Strength (S13), Rewards (S14), Academic ability (S2), Motivation preference (S3), Years qualified as an expert (S4), Morality (S6), and Objective (S7). This set of factors reflects complicated situations. The “dependent factors” belonged to the second cluster (weak driving, strong dependence power) and ranked highest in the importance levels of the ISM-based hierarchical model. Two factors were assigned: Expertise (S1) and the number of bid evaluations (S5). Their dependence indicates that they depended on other factors to be resolved during the bid-evaluation expert performance. Therefore, bid-evaluation experts should consider all other factors to achieve the dependent factors and retain superior expert performance. The third cluster specifies the “linkage factors” (strong driving and dependence power). Any change occurring to these factors would significantly impact other factors in return. No factors correlated with this category in our study, implying that none of them had strong driving and dependence power. The “independent factors” (strong driving, weak dependence power) in the fourth cluster formed the foundation of the ISM hierarchical model and should be given priority. The two factors in this set, Situation perception (S8) and Supervision system (S9), are vital, and bid-evaluation experts and policymakers should focus on them for superior bid-evaluation expert performance.

## Findings and discussion

It is important to enhance expert performance in bid evaluation. The derived model reflects the relationships among expert performance factors in bid evaluation practices.

### A holistic picture of expert performance factors

The two dimensions of internal cognition and the external environment were independent through theoretical deduction. In the PES, the interaction between the “perceptions” and “affordance” of experts is manifested as expert performance, which feeds back or verifies internal cognition and external environment, forming a “closed system” (Figure 1). However, in the context of engineering, 14 factors affect expert performance, seven of which are from the internal cognitive dimension and seven from the external environmental dimension. We could observe that the two-dimensional factors were scattered on four levels (Figure 3). Inherent cognitive factors influenced each other and were scattered at the 1–3 level. Most of them had strong dependence and were driven and influenced by external environmental dimension factors. Level 2–4 was PES, with perception (e.g., body perception) and supply (e.g., the regulatory system) at the lowest end, the most important driving force in the structural system that influences other factors to work together.

The three factors under the heading of the external environment, namely pressure, natural environment, and working distance, together with expertise and bid evaluation times (internal cognitive dimensions), were at the first level of the hierarchical structure system and were the most direct influencing factors. However, the three factors of the environmental dimension existed independently of any other factors, leaving the structure in an open form. These phenomena reflect the organic integration of memory and ecological cognition theories and completely describe the factors affecting expert performance in engineering. These influencing factors differ from the characteristic cognitive ability mentioned in the ecological dynamics theory of sports decision-making with the help of regularity and universality (Araújo et al., 2006). For expert-performance factors with engineering characteristics, inherent cognition has a dynamic influence on expert performance under the constraint of a unique external environment. In contrast, the external environmental factors are more strongly driven or have a direct and independent influence.

### Hierarchy of expert performance factors

In the bid evaluation, considering independent factors (strong driving and weak dependence power from cluster four), situation perception reflects the experts’ feelings regarding the environmental properties, including seating, air humidity, and physical comfort. Negative feelings will hinder expert performance as perception, cognition, and behavior are integrated processes. The nature and type of cognition are influenced by the interaction between the body and the external environment. For example, situation perception, relating to concepts such as fatigue (Orazbayev, 2017), is a hidden expert-performance-influencing factor that few researchers focus on. The perception of “affordance” has a dynamic quality and can change (Fajen et al., 2009). Improved working conditions can maintain enhanced situation perceptions and effectively obtain sustainable and superior expert performance. The other independent factor with strong driving power, a sustainable supervision system rather than self-restriction, is also confirmed to improve expert performance. Environmental properties directly inform experts about what they can and cannot do in a performance context (Michaels, 2000). In essence, the confluence of constraints and perception determines the stability and instability of expert performance.

At level III of the ISM, the factors of morality, objective, and strength precede motivation preference (Figure 3, Level II), while motivation preference, academic ability, and years qualified as an expert (all level II) affect each other bilaterally. Regarding the level II factor, rewards are influenced by the factor of strength. Therefore, these factors with higher driving power in the autonomous cluster (such as Strength) can impact each other. Therefore, their promotion should be given priority in attaining superior expert performance. In contrast, the factors with higher dependence on power are influenced by other factors. Consistent with current studies, academic ability, motivation preference, and years qualified as an expert (Figure 3, Level III) are key factors determining expert performance as acquired skills and experience in bid evaluation.

More factors from inherent cognition and the external environment are plotted at the top of the four-level diagram (Figure 3, Level IV), indicating that factors from the two dimensions collectively determine expert performance. These factors include the inherent cognition dimension (expertise, number of bid evaluations) and the external environment dimension (stress situations, natural environment, and distance).

Previous studies have demonstrated that expertise and exceptional performance are highly reproducible regarding inherent cognition. There is no doubt that unfailed successful learning is necessary for experts to achieve an improved or higher level of performance (Ericsson and Lehmann, 1996). In this regard, of all the factors at the top level, expertise and the number of bid evaluations, both belonging to the inherent cognition dimension, are viewed as the most natural elements influencing expert performance. The other factors: stress situations, natural environment, and distance, which belong to the external environment, are isolated and have no relationship with other factors. This phenomenon shows that the factors affecting experts’ performance are in an open system, and some external environmental factors will directly impact them in some situations. Especially in the bid evaluation process, the pressure from peers or the physical impact of the natural environment on experts (e.g., fatigue caused by long-distance travel to the workplace or bad weather) will make expert performances deviate.

### Key expert performance factors

The situation perception and supervision system factors (Figure 3, Level IV) may forego morality, objective, and strength (Figure 3, Level III), determining expert performance in the bid evaluation. These are key issues to be addressed to realize superior expert performance. Moreover, Ericsson and Lehmann (1996) showed that situation perception affects labor intensity, whereas a strict supervision system initiates moral restraint. Thus, morality and some other factors (including procedural fairness, technical competence, and general reputation) impact and interact with expert performance. Still, illegalities often occur despite the vital importance of limiting moral deviation during expert behavior. For instance, bid-evaluation experts and bidders collude to manipulate the bidding of private customers (forcing the client to pay high prices). In addition, they colluded on certain municipal contracts, causing huge economic losses to New York taxpayers (TargetedNewsService, 2018). It has been reported that experts’ antagonistic feelings may hinder objectivity and obscure experts’ fairness (Lindfield and Penny, 2012).

In particular, three environmental constraint factors (stress situation, natural environment, and distance), located in the top layer, are mainly manifested as physical perception in the natural environment. They do not participate in the PES due to no correlation with any factor in internal cognition and other factors in the external environmental dimension. They make the system open, and these factors show that they directly, solely, and openly affect expert performance in engineering. This contrasts with the traditional information processing methods for decision-making in the “open system.” When decision-makers calculate and select options in the psychological or neural model, the influence of more uncertain external environment factors should be considered to maximize performance effectiveness.

### Implications

Our results show that inherent cognitive and external environmental factors are characterized by high dependence and strong driving power, respectively (Figure 4). For example, Figure 3 Level IV shows that situation perception and supervision system (external environment dimension) are antecedent variables driving expert performance factors and should not be precluded first. However, the classification of factors (Figure 4) shows that morality and objective (internal cognitive dimension) also have higher driving and lower dependence power. This finding suggests that these two special factors bridge the inherent cognitive and external environmental dimensions. Moreover, morality and objectivity (Figure 3, Level III), controlled by the supervision mechanism (Figure 3, Level IV), influence inherent cognition dimension factors.

We confirmed the relationship and hierarchical structure of the two dimensions, suggesting that environmental constraint factors other than inherent cognition contribute to individual expert performance differences in bid evaluation. Furthermore, they show that external environmental factors strongly influence the externalization of inherent cognition. At a practical level, our findings on the two dimensions affirm existing research, supporting the notion that expert performance depends on professional knowledge and the environment in engineering. Furthermore, the findings also provide a basis for exploring ecological cognition based on the idea that the “brain–body-environment system is embedded and embodied.”

## Conclusion

Several uncertainties and factors of expert performance in bid evaluation make its improvement to be a complicated matter. An individual’s performance in a domain is determined by multiple interactions between experience, training, and biological factors. This study produced a comprehensive list of expert performance factors. It identified and examined two dimensions of factors occurring in bid evaluation, and the results indicated that external environmental factors are prominent for expert performance. Furthermore, we also found evidence of cognition-environment interactions, which revealed environmental effects as the most important driving factors of expert performance in bid evaluation. The suggestion is that external environmental factors drive expert performance to change through the role of practice. Thus, poor expert performance can be resolved by fostering a friendly work environment for bid evaluation experts.

Morality and objective can be ranked as factors of ensuring expert performance in bid evaluation. Expert performance should be promoted by driving power factors such as academic ability, motivation preference, and years of qualification. Meanwhile, situation perception and supervision systems are deemed to form the hierarchical foundation, suggesting the need for an eco-friendly environment and enhancement of supervision intensity for good situation perception to support expert performance. In summary, the study sheds some light on the influence relationship and driving relationship among the factors of expert performance to reveal the interaction mechanism between inherent cognition and the external environment. It also extends the concept of expert performance from cognitive science to management science.

Although the study obtained findings on expert performance in bid evaluation, more factors related to the bid evaluation context should be detected to prevent deviations. Furthermore, our research was based on the experience and opinions of interviewees, of which bias and prejudice are unavoidable. Besides, the factors’ interactive relationships have not been quantitatively examined and can be further investigated in future research. Finally, expert performance involves many cognitive–psychological activities. Therefore, it is expected to investigate these activities in future studies to promote expert performance in the engineering context.

## Data availability statement

The original contributions presented in the study are included in the article/supplementary material, further inquiries can be directed to the corresponding author.

## Author contributions

KY and LW: conceptualization. WW: supervision. LW: writing–original draft preparation. KY and YL: writing–review and editing. All authors contributed to the article and approved the submitted version.

## Funding

This work has been supported by National Natural Science Foundation of China (71871033) and Chunhui Project of National Ministry of Education (RZ1900011238).

## Conflict of interest

The authors declare that the research was conducted in the absence of any commercial or financial relationships that could be construed as a potential conflict of interest.

## Publisher’s note

All claims expressed in this article are solely those of the authors and do not necessarily represent those of their affiliated organizations, or those of the publisher, the editors and the reviewers. Any product that may be evaluated in this article, or claim that may be made by its manufacturer, is not guaranteed or endorsed by the publisher.
